# A same day α-synuclein RT-QuIC seed amplification assay for synucleinopathy biospecimens

**DOI:** 10.1038/s44328-024-00023-w

**Published:** 2025-02-11

**Authors:** Sabiha Parveen, Parvez Alam, Christina D. Orrù, Sarah Vascellari, Andrew G. Hughson, Wen-Quan Zou, Thomas G. Beach, Geidy E. Serrano, David S. Goldstein, Bernardino Ghetti, Giovanni Cossu, Giada Pisano, Beatrice Pinna, Byron Caughey

**Affiliations:** 1 Laboratory of Neurological Infections and Immunity, Rocky Mountain Laboratories, National Institute of Allergy and Infectious Diseases, National Institutes of Health, Hamilton, MT USA; 2 Department of Biomedical Sciences, University of Cagliari, Cagliari, Italy; 3 Departments of Pathology and Neurology, Case Western Reserve University School of Medicine, Cleveland, OH USA; 4 Institute of Neurology, Jiangxi Academy of Clinical Medical Sciences, The First Affiliated Hospital, Jiangxi Medical College, Nanchang University, Nanchang, Jiangxi Province China; 5 Banner Sun Health Research Institute, Sun City, AZ USA; 6 Autonomic Medicine Section, National Institute of Neurological Disorders and Stroke, National Institutes of Health, Bethesda, MD USA; 7 Department of Pathology and Laboratory Medicine, Indiana University School of Medicine, Indianapolis, IN USA; 8 S. C. Neurology and Stroke Unit, AOBrotzu, Cagliari, Italy

**Keywords:** Biochemistry, Biological techniques, Biomarkers

## Abstract

Parkinson’s disease (PD), dementia with Lewy bodies (DLB), and other synucleinopathies are characterized by the accumulation of abnormal, self-propagating aggregates of α-synuclein. RT-QuIC or seed amplification assays are currently showing unprecedented diagnostic sensitivities and specificities for synucleinopathies even in prodromal phases years in advance of the onset of Parkinsonian signs or dementia. However, commonly used α-synuclein seed amplification assays take ≥48 h to perform as applied to patients’ diagnostic biospecimens. Here, we report the development of a faster α-synuclein RT-QuIC assay that is as analytically sensitive as prior assays of this type, but can be completed in ≤12 h for brain, skin, and intestinal mucosa, with positive signals often arising in <5 h. CSF assays took a few hours longer. Our same-day α-synuclein RT-QuIC (sdRT-QuIC) assay should increase the practicality, cost-effectiveness, and throughput of measurements of pathological forms of α-synuclein for fundamental research, clinical diagnosis, and therapeutics development.

## Introduction

Neurodegenerative disorders are caused by misfolding, aggregation, and accumulation of proteins in the brain^1–4^. The molecular pathology underlying synucleinopathies such as Parkinson’s disease (PD) and dementia with Lewy bodies (DLB) is characterized by misfolding and aggregation of abnormal α-synuclein (α-syn)^5^. Synucleinopathology is driven by α-syn assemblies that propagate through seeded polymerization and intercellular spreading^6–8^. In PD, motor symptoms appear after a long prodromal phase, followed by dementia in a majority of the patients at later stages. In DLB, cognitive deficits manifest earlier and progress faster than in PD^9^. DLB is the second most common cause of dementia after Alzheimer’s disease (AD). Lewy bodies (LBs) and Lewy neurites containing α-syn aggregates in neurons are associated with PD and DLB. Due to overlapping symptoms among various synucleinopathies and other neurodegenerative disorders, specific diagnosis at an early stage can be difficult^10–12^. However, magnetic resonance imaging of the brain (MRI brain), dopamine transporter (DaT) scans, PET scans, and blood work can help to rule out other medical conditions and support diagnoses of synucleinopathy^13,14^.

Real-time quaking-induced conversion (RT-QuIC) assays are seed amplification assays (SAAs) that exploit the seeded polymerization mechanism of various pathological protein assemblies to allow their ultrasensitive detection in tissues, biofluids, and contaminated surfaces. RT-QuIC assays provide a pathology-specific biomarker for neurodegenerative disorders^15,16^. Small amounts of misfolded proteins with seeding activity in biospecimens can be amplified by many orders of magnitude in vitro using recombinant α-syn as substrate in multi-well plates with thioflavin T (ThT) fluorescence-based detection. RT-QuIC assays have detected pathological α-syn aggregates in a variety of biospecimens including brain^17^, cerebrospinal fluid (CSF)^18^, olfactory mucosa^19,20^, submandibular gland^21^, blood and serum^22,23^, skin^24,25^, salivary gland^26^, and intestinal mucosa (IM)^27^, potentiating identification of synucleinopathy cases sensitively and specifically in prodromal phases of disease.

Commonly used α-syn RT-QuIC assays developed by us and others can detect seeding activity in diagnostic specimens within an overall ~48 h assay window^28^. Here, we report a faster same-day α-syn RT-QuIC (sdRT-QuIC) assay for various biological specimens, including brain, skin, and intestinal mucosa (IM), that, in most cases, can be completed within ~12 h. CSF α-syn sdRT-QuIC assay took a few hours longer to complete. Moreover, our assay works with equivalent sensitivity and specificity compared to our previous α-syn RT-QuIC-Rapid (RT-QuICR) assay^28^. While preparing this manuscript another study found, as we have, that the addition of non-ionic detergents can reduce RT-QuIC assay times in applications to CSF specimens^29,30^.The availability of these more rapid assays should accelerate, and reduce costs, of α-syn aggregate detection in both clinical and research applications.

## Results

### sdRT-QuIC assay development using brain homogenates

**Fig. 1:**
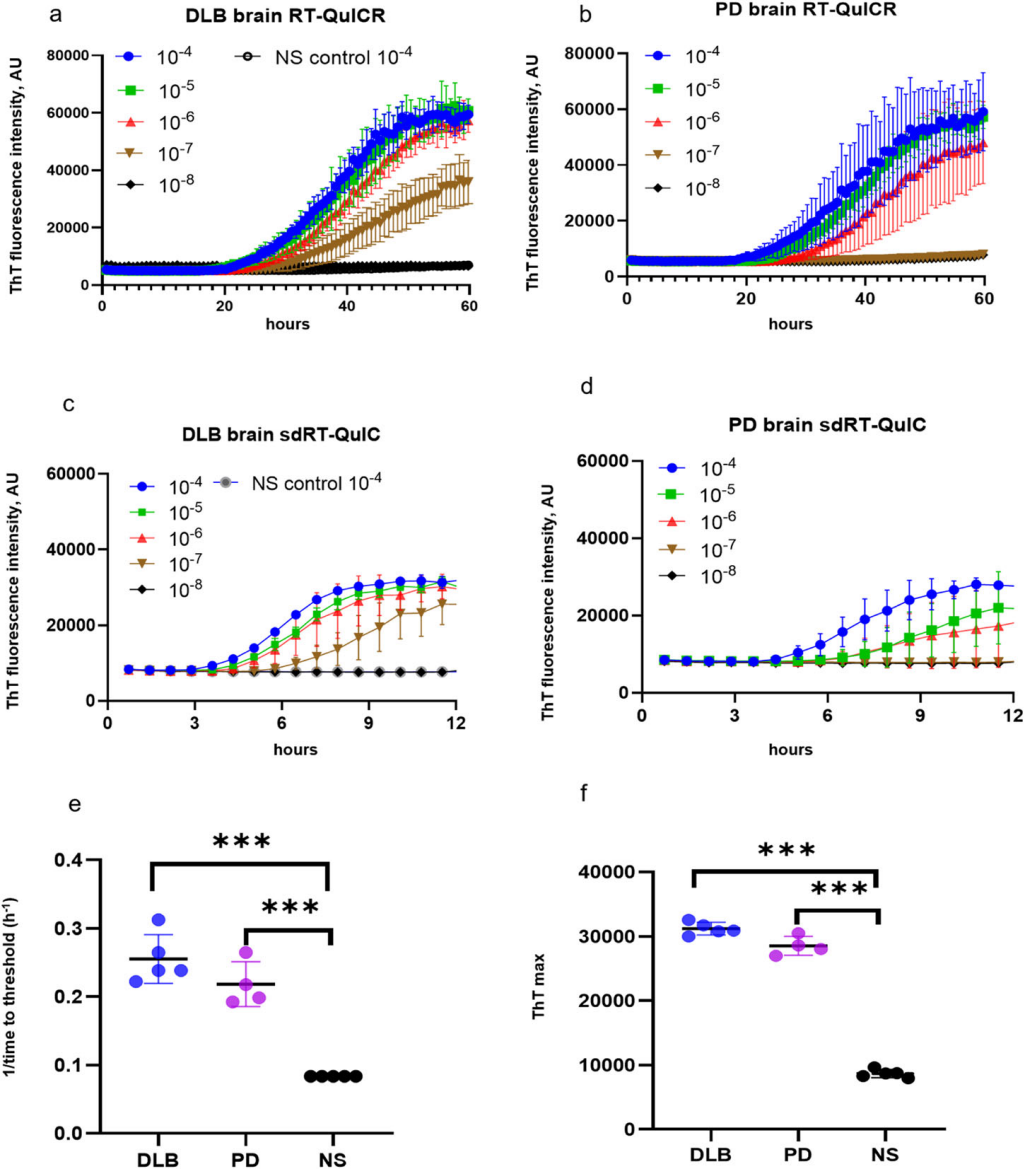
Analysis of the brain using the RT-QuICR (a,b) and sdRT-QuIC (c-f) assays. **a**–**d** End-point dilution analyses of single DLB (**a**, **c**) and PD (**b**, **d**) specimens. ThT fluorescence traces show means ± SD of quadruplicate reactions at the designated dilutions given with respect to the original tissue mass rather than homogenates thereof. Non-synucleinopathy (NS) brain at 10^−4^ dilution was included as a negative control, with the traces shown in (**a**, **c**) also applying to the plots in (**b**, **d**). **e** 1**/**TTT and **f** ThT maxima were obtained using 10^−4^ DLB (*n* = 5), PD (*n* = 4), and NS (*n* = 5) brain dilutions. Data points represent means of quadruplicate reactions from individual cases, and bars represent the mean ± SD of the means from all cases of that type. The same brain specimens were used in these RT-QuICR and sdRT-QuIC assays. Statistical significance is denoted as *p < 0.05, **p < 0.01, ***p < 0.001.

We used recombinant K23Q α-syn mutant as a substrate because we and others have found it to be more stable against spontaneous, unseeded nucleation than wildtype α-syn^28^. Assay conditions with variations in salt (Hofmeister ions as discussed previously^31^), temperature (42 to 50 °C), shaking speed (400 to 700 rpm), α-synuclein monomer concentration (0.09 to 0.12 mg/ml) and detergents (e.g., SDS (0.001 to 0.1%), Triton X-100 (0.001 to 0.2%) were compared to arrive at the current sdRT-QuIC conditions. We focused initially on the brain from DLB (*n* = 5), PD (*n* = 4), and non-synucleinopathy (NS) cases (*n* = 5) and performed end-point dilution analyses to compare seed concentrations and assay sensitivities as described previously^28,32,33^. In such assays, samples are serially diluted (titrated) to determine the dilution at which positive assay responses are lost. When seeded with 10^−4^ dilutions of brain tissue (Note: All subsequent dilution factors are given relative to the original specimen/tissue rather than homogenates thereof), DLB or PD cases gave a rapid increase in ThT fluorescence within 3–5 h in quadruplicate reactions, whereas the NS controls gave no enhancement over 12 h (Fig. 1). With further dilutions of DLB and PD brain homogenates (BH) down to 10^−7^ and 10^−6^ respectively, the quadruplicate reactions became ThT positive within 12 h. Beyond these near-end-point dilutions, smaller proportions of the quadruplicate reactions, if any, became ThT positive within 12 h. With the NS negative controls (*n* = 5), no above-threshold ThT fluorescence was observed when seeded with 10^−4^ to 10^−8^ dilutions. 1/time to threshold (1/TTT or 1/lag time) values at 10^−4^ dilution of the brain were used as an indication of relative protein aggregation rate (Fig. 1e). Mean 1/TTT values for PD and DLB brains were significantly higher than controls consistent with the presence of more seeding activity. A dose dependent decrease in 1/TTT was observed for DLB and PD brains with increasing dilutions. In summary, our final combination of Triton X-100 concentration, temperature, and shaking speed (i.e., sdRT-QuIC) accelerated the assay kinetics by ~4-fold without a disproportionate acceleration of NS control reactions. Other than the faster kinetics, these sdRT-QuIC results were consistent with previous α-syn RT-QuIC assays in terms of sensitivity and specificity, as reported by us and other labs^28,34^.

### sdRT-QuIC assay of the intestinal mucosa (IM) biopsies from PD and NS patients

**Fig. 2:**
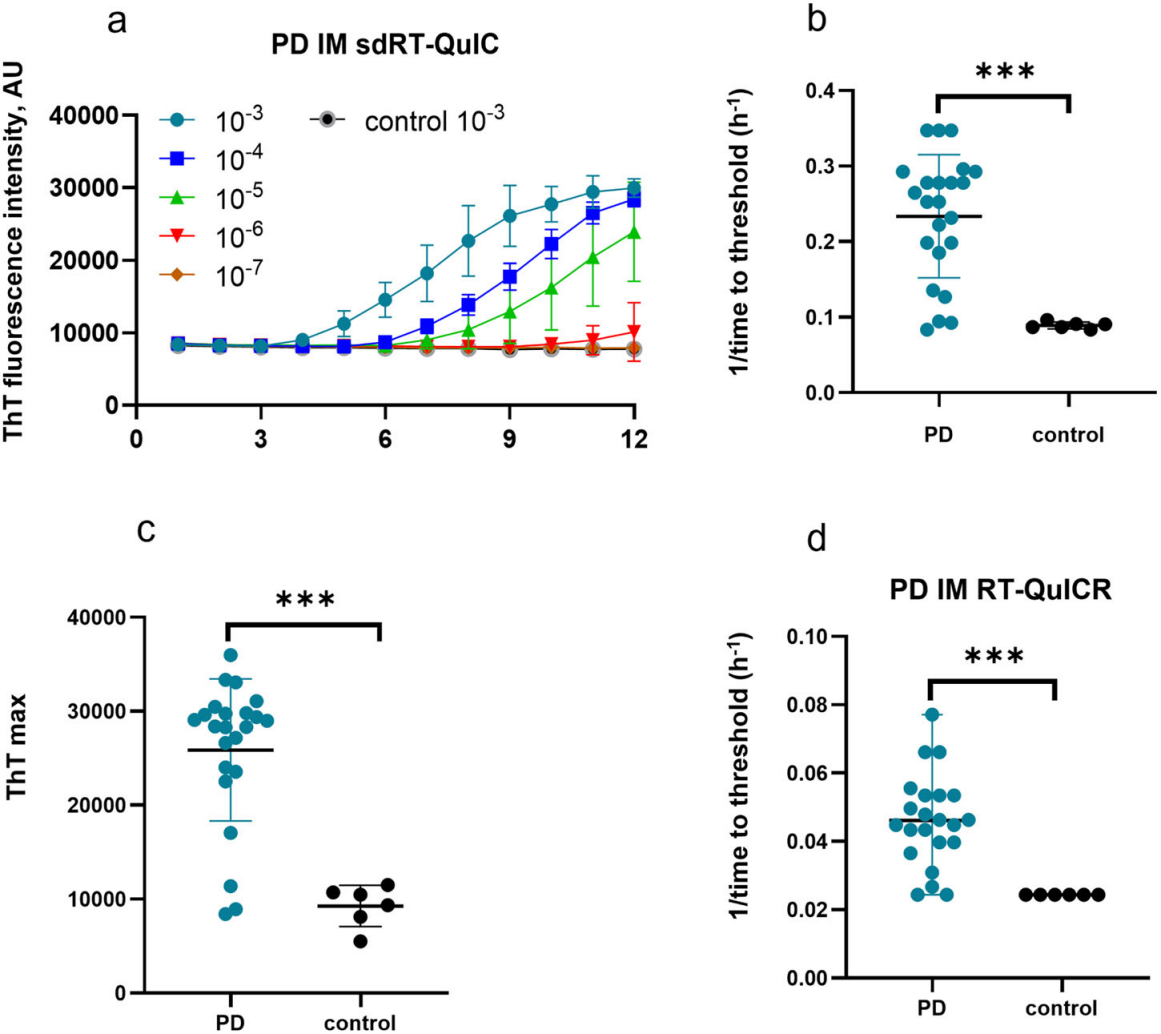
sdRT-QuIC and RT-QuICR analyses of intestinal mucosa (IM) biopsies. **a** Representative end-point dilution analysis of a PD and an NS control IM sample. Data points indicate means ± SD of quadruplicate reactions at the designated dilutions of PD (*n* = 1) and control IMs (*n* = 1). **b** 1**/**time to threshold and **c** ThT maxima obtained from 10^-3^ dilutions of PD (*n* = 23) and NS (*n* = 6) IMs. **d** 1**/**TTT at 10^−3^ dilution of the same panel of PD and NS IMs using the RT-QuICR assay (data summarized from Fig. 3 of ref. ^27^). In **b**–**d**, data points represent means of quadruplicate reactions from individual cases, and bars represent the mean ± SD of the means from all cases. The same IM specimens were used in the RT-QuICR and sdRT-QuIC assays. Statistical significance is denoted as *p < 0.05, **p < 0.01, ***p < 0.001.

To further evaluate the diagnostic potential of our sdRT-QuIC assay, we analyzed IM biopsies obtained from PD (*n* = 23) and NS control (*n* = 6) patients. IM samples were collected as described previously and blinded to the sdRT-QuIC analyst. End-point dilution analysis was performed on each of the PD IM samples with data from one PD and one NS controls shown in Fig. 2a. Like DLB and PD brains, PD IM TTT were 3–5 h at 10^−3^ dilution in majority of samples. The seeding activity was also detected at 10^−4^ dilution of IMs in all quadruplicate sdRT-QuIC reactions. One PD IM was ThT negative at 10^−3^ dilution but became positive on further dilution, consistent with our previous report using the RT-QuICR assay^27^; this was likely due to sample matrix inhibition at the higher concentration. All our NS control quadruplicates remained negative at the 10^−3^–10^−5^ dilutions that were tested (Fig. 2). The TTT and ThT max values obtained using 10^−3^ dilutions were significantly different between PD IMs and NS controls (Fig. 2b, c). In agreement with our previous study, IM samples 6, 7, and 21 contained less seeding activity than the other PD IMs (lowest 3 PD data points in Fig. 2c, d). Overall, other than the faster reaction kinetics (compare Fig. 2b, d; data in d were adapted from Fig. 3 of ref. ^27^ for comparative purposes), the sdRT-QuIC results were comparable to previous results using our RT-QuICR assay in terms of sensitivity and specificity^27^.

**Fig. 3:**
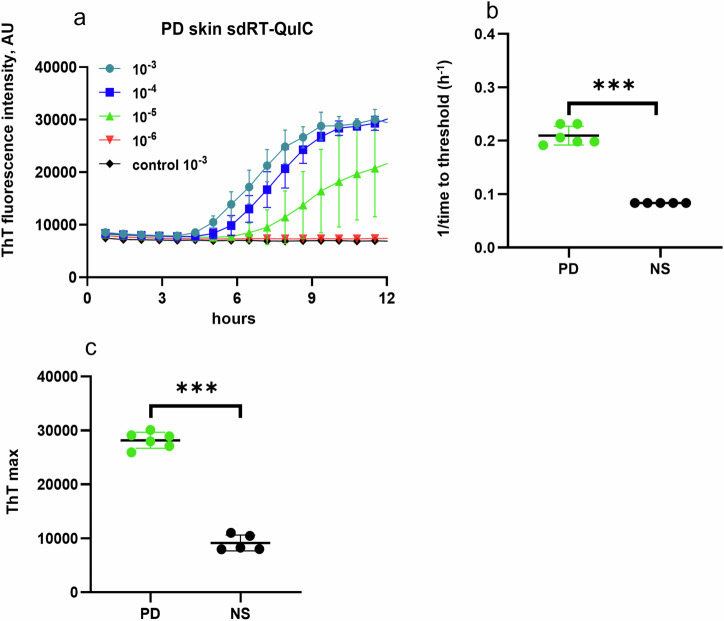
sdRT-QuIC analysis of skin. **a** End-point dilution analysis of skin samples from representative PD (*n* = 1) and NS (*n* = 1) cases. Data points indicate means ± SD of quadruplicate reactions seeded with the designated dilutions of the PD skin samples. **b** 1**/**TTT and **c** ThT maxima obtained from 10^−3^ dilutions of PD skin (*n* = 6) and NS control (*n* = 5) cases. In **b**, **c**, data points represent means of quadruplicate reactions from individual cases, and bars represent the mean ± SD of the means from all cases. Statistical significance is denoted as *p < 0.05, **p < 0.01, ***p < 0.001.

### sdRT-QuIC analysis with skin samples

As with brain and IM specimens, sdRT-QuIC discriminated postmortem skin samples from PD and NS cases within 12 h. PD skin samples gave positive ThT responses in all quadruplicates at 10^−3^ to 10^−4^ dilutions, while only a subset of quadruplicates was ThT positive with further dilutions (Fig. 3). NS skin samples remained negative for all dilutions tested (10^−3^–10^−5^) (Fig. 3). The differences between the mean PD and NS skin 1/TTT and ThT maximum values were significant.

### sdRT-QuIC analyses with antemortem PD CSF

**Fig. 4:**
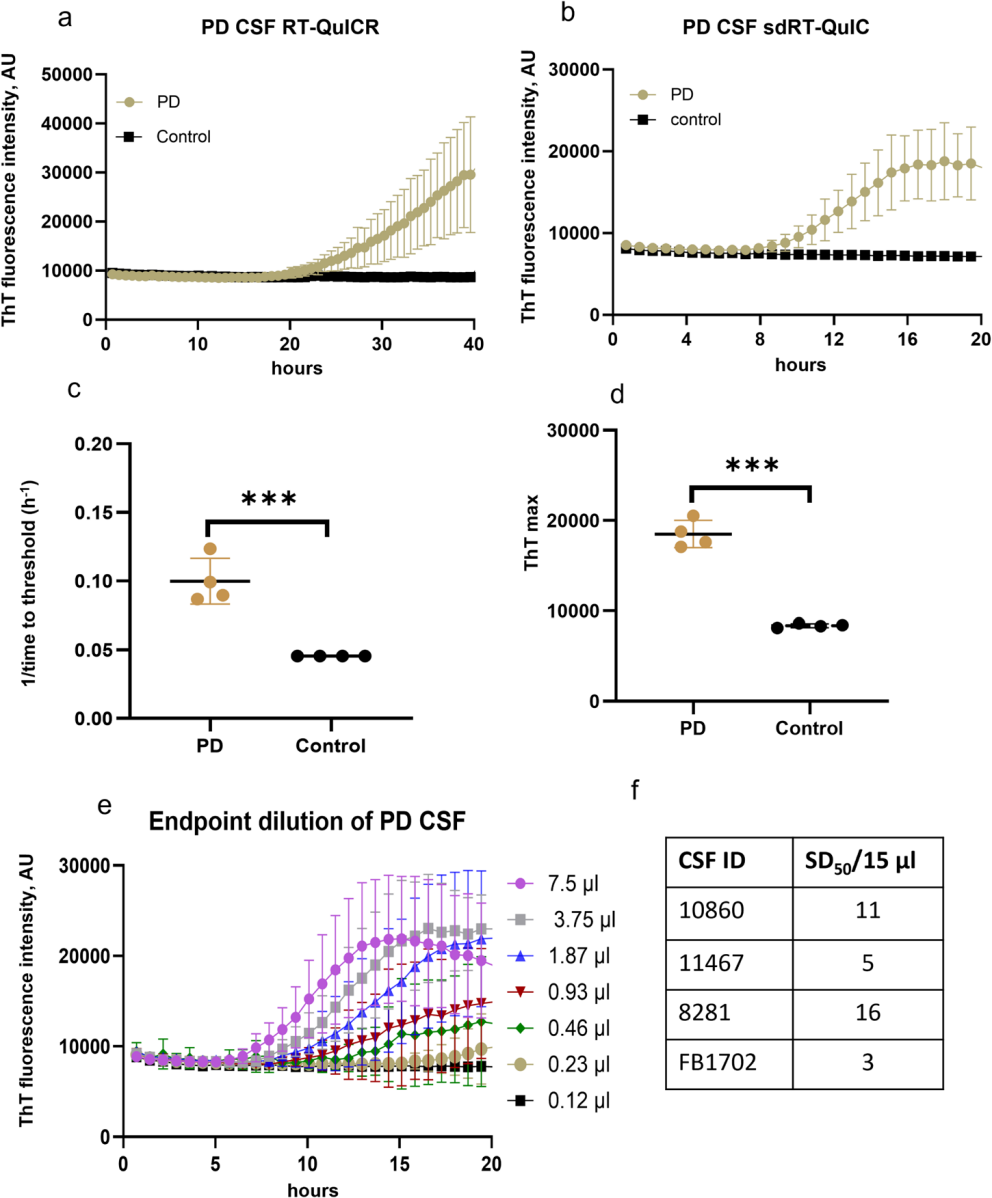
sdRT-QuIC analysis of CSF. Representative analyses of single PD and NS control CSF specimens using **a** RT-QuICR and **b** sdRT-QuIC. Traces show means ± SD of ThT fluorescence from quadruplicate reactions. **c** 1**/**TTT and **d** ThT maxima within 20 h of sdRT-QuIC reactions seeded with PD (*n* = 4) and NS (*n* = 4) CSFs. The same CSF samples were used in the respective RT-QuICR and sdRT-QuIC assays. Data points represent means of quadruplicate reactions from individual cases, and bars represent the mean ± SD of the means from all cases. **e** End-point dilutions of a PD CSF by sdRT-QuIC. Each trace represents the mean ± SD ThT fluorescence of quadruplicate wells. **f** log SD_50_/15 µl values for PD CSFs (*n* = 4). Statistical significance is denoted as *p < 0.05, **p < 0.01, ***p < 0.001.

We also applied our sdRT-QuIC assay to antemortem CSF specimens. All PD CSFs (*n* = 4) gave positive RT-QuIC responses within 10–12 h, whereas NS controls remained negative. There was clear discrimination between PD and NS CSFs in terms of ThT kinetics, 1/TTT, and ThT max (Fig. 4). However, CSF-seeded reaction kinetics were generally slower than observed with the other biospecimens, with lag phases averaging ~10 h (i.e., 1/TTT = 0.1 h^−1^). Overall, our sdRT-QuIC assay was sensitive enough to discriminate between CSF from PD and NS control cases. Furthermore, to quantify the seeding activity in PD samples, we performed an end-point dilution analysis. We detected seeding activity in as little as 0.46 µl of PD CSF (Fig. 4e), which was similar to what we have reported for another PD CSF using our RT-QuICR assay^28^.

### Relative α-syn seeding activities in different biospecimens

**Fig. 5:**
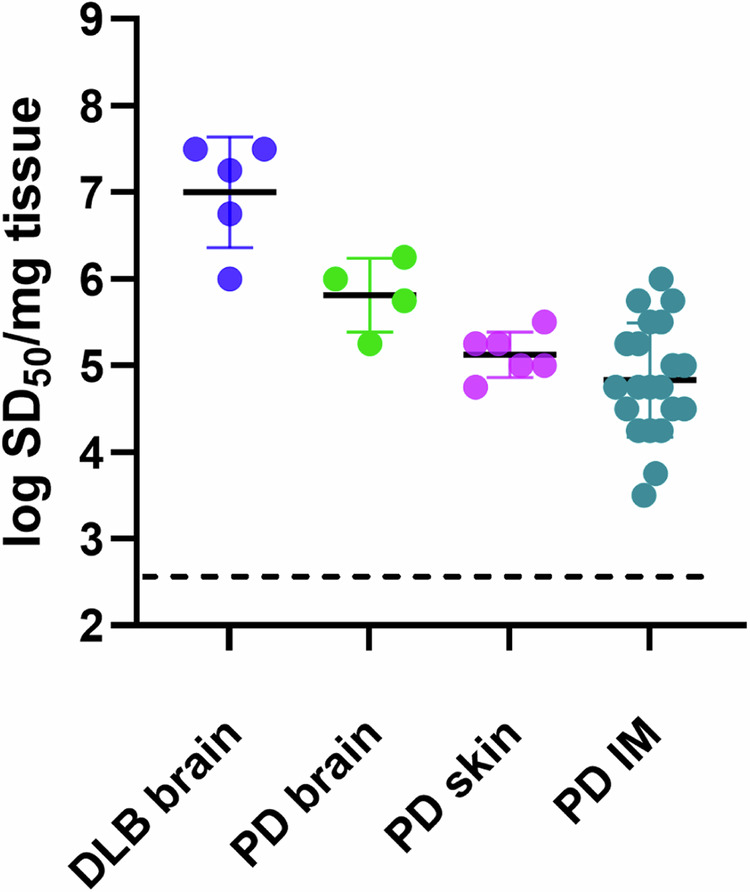
Seed concentration (log SD_50_/mg tissue) determination for DLB (*n* = 5) and PD (*n* = 4) brain, PD skin (*n* = 6), and PD IM (*n* = 23) samples by sdRT-QuIC. Data points represent log SD_50_/mg tissue values calculated from sample dilution series with quadruplicate reactions at each dilution, and bars represent mean ± SD of values from all cases.

Using a modified Spearman–Kärber algorithm, we compared seed concentrations in different types of biospecimens by estimating the amount of sample containing enough seeding activity to yield positive (above-threshold) responses in 50% of technical replicate reactions, i.e., the 50% seeding dose or SD_50_^35^. SD_50_ is analogous to the 50% lethal dose (LD_50_) that is commonly estimated in titrations of pathogen titers in animal bioassays, and back calculations can determine the SD_50_ concentration per unit of undiluted sample. The average log SD_50_/mg of tissue values for DLB brains, and PD brains were 7.0 and 5.8, respectively. Similarly, log SDS_50_/mg values for PD skin and PD IM samples were 5.3 and 5.1. The average SDS_50_/15 µl for CSF was 9 (0.95 log) (Figs. 4f, 5). These sdRT-QuIC log SD_50_/mg were consistent with those obtained previously with RT-QuICR^27,28^.

## Discussion

Given that a diverse set of neurodegenerative disorders involve the accumulation of abnormal α-syn aggregates, it is important to have fast and sensitive assays for such aggregates. α-syn RT-QuIC assays offer detection methods that are more sensitive than, and complementary to, conventional methods such as immunohistochemistry, immunoblotting, and ELISA. This added sensitivity enhances the ability to detect α-syn aggregates in both primary and secondary synucleinopathies. The earliest α-syn SAAs, including the first α-syn RT-QuIC by Green et al.^36^ and the α-syn PMCA by Soto et al.^18^, had assay times ranging from 100 h to over 300 h. Sano et al. reported an αSyn RT-QuIC that could detect seeding activity in DLB brain tissues at extreme dilutions in 96 h^17^. Soon thereafter, some of us reported an α-syn RT-QuIC assay requiring only ~48 h^28^. In comparison, our sdRT-QuIC can be completed within ~12 h when assaying brain, skin, and IM specimens under the same assay conditions, while maintaining sensitivity comparable to our previously reported assay. For reasons that remain unclear, CSF specimens gave longer lag phases (mean = ~10 h) than the other biospecimens, but the CSF-seeded kinetics were nonetheless much faster than our previous assay (Fig. 4).

Influential factors in optimizing the assay speed and performance included temperature, salts, beads, shaking speed, and detergents can modulate the speed of RT-QuIC reactions^29,37,38^. However, the exact mechanisms behind the effects of these factors are not clear and are likely complex^28^. It seems likely that elevated temperature increases collision between seeds and monomers, and shaking helps with reaction mixing and fragmentation of growing fibrils to generate more seeding surfaces. Triton X-100 might improve the distribution of seeds and/or the conformation of monomers to predispose them to conversion. In any case, reduced assay times of sdRT-QuIC should enhance the practicality and cost-effectiveness of SAA applications in both research and clinical settings. Importantly, in our testing of synucleinopathy biospecimens, sdRT-QuIC sensitivity and specificity, as well as the relative seed concentrations measured in different types of biospecimens, were comparable to results obtained with previously reported SAAs^27,28^.

A limitation of this work is that was performed with a limited number of biospecimens to provide an initial proof-of-principle basis for many future applications. Clearly, testing on much larger and more complex sample sets will be required to better establish the sdRT-QuIC assay’s utility in clinical and research applications.

In summary, we have developed a faster α-syn RT-QuIC assay for ultrasensitive detection of pathological synuclein seeds in synucleinopathy brains, skin, and IM samples with an overall assay time of ~12 h. CSF assays required slightly longer but still less than 1 d. Further optimizations will be needed to make CSF analyses comparable in speed to those of the other biospecimens, and to establish sdRT-QuIC applications to more accessible biospecimens such as blood, tears, and saliva. Nonetheless, our same-day RT-QuIC assay should facilitate measurements of α-syn aggregates in diagnostics, high throughput screening of drug candidates, clinical trials, and fundamental research.

## Methods

### Expression and purification of α-syn

The α-syn protein was purified in-house using our previous method^28^ and the modified method described below. Briefly, 5 mL of LB media containing 50 μg/mL kanamycin were inoculated from a glycerol stock of E. coli bacteria containing vectors for K23Q α-syn protein. Following 5–6 h of incubation with continuous 225 rpm agitation at 37 °C, 1 L of the auto-induction media containing 50 μg/mL kanamycin was prepared, and the 5 mL starter culture was added. The cells were grown in a shaking incubator at 37 °C with shaking at 225 rpm, overnight. The next day, cells were harvested by splitting the 1 L culture into four 250 mL conical tubes and centrifuging at 3750 × *g* at 4 °C, for 12 min. The cell pellets were each suspended in 30 mL of 20 mM Tris pH 7.4, containing Benzonase Nuclease (EMD 70746-3 diluted 1:1000) and Roche Protease inhibitor tablet, transferred to a fresh 50 mL tube, mixed well with a 25 mL serological pipette, and then sonicated (4 × 45 s with 15 s rest) at a power setting of 45% using a probe sonicator (Sonics VibraCell, Newtown CT, USA) to lyse the bacterial cells. The 50 mL tubes were placed in a boiling water bath for 20 min to denature α-syn in the lysate. The lysates were then centrifuged at 9000 × *g* for 60 min at 20 °C. Supernatants were collected and combined from all four tubes. About 50 mL of 20 mM Tris pH 7.4 was added to the supernatant and filtered through a 0.22-μm vacuum filter. Next, the supernatant was loaded onto a 5 mL Ni-NTA column (Qiagen) on an Äkta Pure chromatography system (GE) and washed with 20 mM Tris, pH 7.5 at room temperature. The column was further washed with 50 mM imidazole, 20 mM Tris, and pH 7.5, which generated a peak in the 280 nm absorption elution profile that was not collected. A linear gradient from 50 to 500 mM imidazole in 20 mM Tris, pH 7.5, was applied, and a peak was collected between 150 and 375 mM imidazole. This peak was then loaded onto a Q-HP column (GE) and washed with 20 mM Tris, pH 7.5. The column was further washed with 100 mM NaCl, 20 mM Tris, and pH 7.5. A linear gradient up to 500 mM NaCl in 20 mM Tris, pH 7.5, was performed, and a peak was recovered between 300 and 350 mM NaCl. The protein was filtered through a 0.22-μm filter and dialyzed against prechilled PBS overnight at 4 °C using a 3.5 kDa MWCO dialysis membrane. The next day, the protein was moved into fresh prechilled PBS for another 4 h of dialysis. Protein solutions were filtered through a 0.22-μm filter, and the protein concentration was determined with a UV–VIS spectrophotometer using a theoretical extinction coefficient at 280 nm of 0.36 (mg/mL)^−1^ cm^−1^. The protein was then aliquoted and stored at −80 °C until further use.

### Sample collection

Brain (DLB, PD), skin (PD), IM (PD), CSF (PD), and respective control samples used in this study were collected as described in our previous studies^24,27,28,39^. Descriptions of samples used in this study are given in Tables 1–4.

**Table 1:** List of synucleinopathy and control brain samples used in this study

ID	Diagnosis/cause of death	Age^a^	Sex (M/F)
4349	PD	61	M
4762	PD	80	M
AN12945	PD	71	M
AN01502	PD	72	F
4699	DLB	72	M
2006-016	DLB	81	M
2004-016	DLB	80	M
2006-005	DLB	73	M
2006-020	DLB	71	M
	**Controls (non-synucleinopathy)**		
2008-049	CBD	51	F
2005-059	CBD	65	M
5451	Hypertensive atherosclerosis heart disease	57	F
5511	Pneumonia	80	F
5919	Drowning	63	M

^a^at time of death.

**Table 2:** List of PD and control skin autopsy samples used in this study

ID	Diagnosis	Motor UPDRS score_months prior to death	Age^a^	Sex (M/F)
17-54	PD	19	79	F
15-57	PD	6	90	M
12-65	PD	15	74	M
16-55	PD	11	81	F
18-31	PD	39	79	F
13-07	PD	9	88	F
	**Controls (non-synucleinopathy)**			
08-55	ND	-	71	M
11-12	AD	-	76	F
14-48	AD	-	73	M
17-60	AD	-	82	M
21-53	AD		79	M

*UPDRS* Unified Parkinson’s Disease Rating Scale.

*PD* Parkinson’s disease.

*ND* Non-disease.

*AD* Alzheimer’s disease.

^a^at time of death.

**Table 3:** List of PD and control intestinal mucosal biopsy samples used in this study

ID	Age^a^	Sex (M/F)	Disease duration
IMPD1-2	73	F	6
IMPD2	65	M	25
IMPD3-2	75	M	11
IMPD4	65	F	15
IMPD5	65	F	10
IMPD6	76	F	14
IMPD7	58	F	11
IMPD8	58	M	15
IMPD9	66	M	17
IMPD10	70	M	12
IMPD11	70	F	15
IMPD12	69	F	12
IMPD21	58	F	14
IMPD 25	20	F	20
IMPD15	57	F	20
IMPD16	69	F	12
IMPD17	56	M	8
IMPD18	77	M	6
IMPD22-1	56	F	19
IMPD23-1	55	M	13
IMPD24-1	77	M	20
IMPD26-2	58	F	8
IMPD27-2	74	M	13
IM19-2	42	F	
IM20-1	59	F	
IM29-2	72	M	
IM30-2	53	F	
IM30-1	53	F	
IM31-2	62	F	

IMPD and IM represent biopsies from PD and healthy controls.

^a^At time of biopsy

**Table 4:** List of synucleinopathy and control CSF samples used in this study*

ID	Diagnosis	Age	Sex (M/F)
10860	PD	72.6	M
11467	PD	74.7	F
8281	PD	56.6	F
FB1702	PD	69.6	M
	**Controls CSF (non-synucleinopathy)**		
8469	PD Risk factors, LBD-	39.2	F
11357	Healthy volunteer	39.2	M
7423	PD risk factors, LBD-	55	F
8171	PD risk factors, LBD-	62.7	F

^*^Data were included about RT-QuIC in CSF samples from some subjects in other collaborative studies with intramural NINDS. Those studies are focused separately on applying CSF RT-QuIC to distinguish among forms of central neurodegeneration in cross-sectional studies or predict subsequent symptomatic central neurodegeneration in longitudinal studies.

### Brain homogenate preparation

Brain homogenates (BH; 10% w/v) were prepared by homogenizing the tissue in PBS using a bead beater for 1 min at maximum speed with zirconia beads (1 mm in diameter). The homogenate was then spun at 2000 × *g* for 2 min at room temperature. The supernatants were collected, and aliquots were stored at −80 °C until further use. For α-syn RT-QuIC testing, homogenates were serially diluted in PBS.

### Skin homogenate preparation

Skin homogenates (SH; 10% w/v) were prepared by homogenizing the tissue in TBS homogenization buffer containing 2 mM CaCl_2_, 1 mg/mL Collagenase A (Roche Cat # 10 103586001) using a Bead Beater (Biospec Products; 11079110z) for 1 min at maximum speed. The homogenates were then incubated in a TBS homogenization buffer at 37 °C for 2 h and then homogenized again for 1 min at maximum speed in a Bead Beater. The homogenates were then spun at 2000 × *g* for 2 min at room temperature, and the supernatants were transferred to a new tube and stored at −80 °C until further use in α-syn RT-QuIC assay.

### Intestinal mucosa homogenates preparation

IM samples were homogenized as discussed in our previous paper^27^. After thawing, IMs were washed 3x in PBS, weighed, and homogenized at 10% (w/vol) in ice-cold homogenization buffer containing 1x PBS, 0.1% Triton X-100, 150 mM NaCl, 5 mM of ethylene-diamino-tetraacetic acid (EDTA), and complete protease inhibitor without EDTA (Roche). Homogenization was performed using 1 mm zirconia beads in a mini-Bead Beater, with four 1-min rounds at maximum speed, with cooling between each round. The homogenates were then centrifuged for 5 min at 500 × *g*. Aliquots of the supernatants were stored at −80 °C until further use.

### Original RT-QuICR assay

RT-QuIC assays were performed in black 96-well plates with a clear bottom (Nalgene Nunc International, Rochester, NY). Wells were loaded with six beads, 0.8 mm in diameter (molecular biology grade silica beads, OPS Diagnostics, Lebanon, NJ). For reactions seeded with brain, skin, and IM homogenates, 2 μL of the indicated dilutions of homogenate were added to wells containing 98 μL of the reaction mix with a final concentration of 40 mM phosphate buffer (pH 8), 170 mM NaCl, 0.1 mg/ml recombinant α-syn, and 10 μM of ThT. For CSF seeded reactions 85 µL of a reaction mixture was mixed with 15 µL of CSF, to give final assay concentrations of 40 mM phosphate buffer (pH 8.0), 170 mM NaCl, 10 μM thioflavin T (ThT), 0.0015% sodium dodecyl sulfate, and 0.1 mg/mL of recombinant α-syn, which was purified as described previously^28^. The α-syn was filtered through a 100 kDa molecular weight cutoff filter immediately prior to use. Reactions were performed in quadruplicate. The plate was sealed with a plate sealer film (Nalgene Nunc International) and incubated at 42 °C in a BMG FLUOstar Omega plate reader (BMG Labtech, Cary, NC) with cycles of 1 min shaking (400 rpm double orbital) and 1 min rest. ThT fluorescence measurements (450 ± 10 nm excitation and 480 ± 10 nm emission; bottom read) were taken every 45 min.

### sdRT-QuIC assay

sdRT-QuIC reactions were performed in black 96-well plates with a clear bottom (Nalgene Nunc International, Rochester, NY). The wells were preloaded with six 0.8 mm in diameter molecular biology grade silica beads (OPS Diagnostics, Lebanon, NJ). For reactions seeded with brain, skin and IM homogenates, 2 μL of the indicated dilutions of homogenate were added to wells containing 98 μL of the reaction mix with final concentration of 40 mM phosphate buffer (pH 8), 150 mM NaCl, 0.1% Triton X-100, 0.1 mg/ml recombinant α-syn and 10 μM of ThT. For CSF-seeded reactions, 85 µL of the reaction mixture and 15 μL of CSF was added per well to give final concentrations of 40 mM phosphate buffer (pH 8.0), 150 mM NaCl, 0.1% Triton X-100, 0.1 mg/mL recombinant α-syn and 10 μM ThT. Reactions were carried out in quadruplicate. The protein was filtered through a 100 kD MWCO filter immediately before use. The plate was sealed with a plate sealer film (Nalgene Nunc International) and incubated at 50 °C in a BMG FLUOstar Omega plate reader (BMG Labtech, Cary, NC) with cycles of 1 min shaking (700 rpm double orbital) and 1 min rest. ThT fluorescence measurements (450 ± 10 nm excitation and 480 ± 10 nm emission; bottom read) were taken every 45 min. Samples were classified as RT-QuIC positive or negative based on criteria similar to those previously described^32^. A sample was considered positive overall when at least two of four replicate wells crossed this calculated threshold.

### Statistical analysis

Student’s *T*-test was used to analyze differences between cases and controls. Variability of the data were presented as mean ± SD. All statistical analyses were performed and plotted using the GraphPad Prism software. Statistical significance was set at *p* < 0.05.

### Ethics approval

Skin samples were obtained postmortem under IRB# NHR-16-16 from the Center for Clinical Research and Technology at Case Medical Center who determined that the use of the samples did not qualify under Federal regulations as “human subject research”. The collection of IM biopsies was approved by the Institutional Ethics Committee (Prot.PG/2017/17817) of the Azienda Ospedaliera Universitaria di Cagliari, Italy. Antemortem CSF samples were obtained from the National Institute of Neurological Disorders and Stroke (NINDS) under a secondary research protocol (000490) approved by the NIH IRB. Brain tissue specimens were obtained postmortem from the Indiana University School of Medicine and the NIH Brain & Tissue repository-California. No additional ethical permission was needed because the samples were taken from deceased, deidentified, consenting individuals. Written informed consent was obtained from patients or their legal representatives for the collection of all specimens (i.e., brain tissue, skin, cerebrospinal fluid, and OM). All samples were deidentified prior to shipping to NIAID for the assays performed in this study.

## Supplementary information

Supplementary Data File 1

## Data Availability

All primary data RT-QuIC data supporting the figures are provided in Supplementary Data File 1.
